# The Regulatory Roles of Polysaccharides and Ferroptosis-Related Phytochemicals in Liver Diseases

**DOI:** 10.3390/nu14112303

**Published:** 2022-05-30

**Authors:** Yijing Ren, Siyue Li, Zixuan Song, Qiuping Luo, Yingying Zhang, Hao Wang

**Affiliations:** Department of Nutrition, Precision Nutrition Innovation Center, School of Public Health, Zhengzhou University, Zhengzhou 450001, China; renyijing1204@163.com (Y.R.); lisiyue911@163.com (S.L.); songzixuan1020@163.com (Z.S.); lqp_mqq@163.com (Q.L.); zyy18614986971@163.com (Y.Z.)

**Keywords:** polysaccharide, phytochemical, ferroptosis, liver injury

## Abstract

Liver disease is a global health burden with high morbidity and mortality worldwide. Liver injuries can develop into severe end-stage diseases, such as cirrhosis or hepatocellular carcinoma, without valid treatment. Therefore, identifying novel drugs may promote liver disease treatment. Phytochemicals, including polysaccharides, flavonoids, alkaloids, and terpenes, are abundant in foods and medicinal plants and have various bioactivities, such as antioxidation, immunoregulation, and tumor killing. Recent studies have shown that many natural polysaccharides play protective roles in liver disease models in vitro and in vivo, such as fatty liver disease, alcoholic liver disease, drug-induced liver injury, and liver cancer. The mechanisms of liver disease are complex. Notably, ferroptosis, a new type of cell death driven by iron and lipid peroxidation, is considered to be the key mechanism in many hepatic pathologies. Therefore, polysaccharides and other types of phytochemicals with activities in ferroptosis regulation provide novel therapeutic strategies for ferroptosis-related liver diseases. This review summarizes our current understanding of the mechanisms of ferroptosis and liver injury and compelling preclinical evidence of natural bioactive polysaccharides and phytochemicals in treating liver disease.

## 1. Overview of Liver Diseases and Polysaccharides

Chronic liver disease (CLD) is an important public health problem in the world, which is a major cause of morbidity and mortality worldwide. There are many types of CLDs, mainly including alcoholic liver disease (ALD), nonalcoholic fatty liver disease (NAFLD), viral hepatitis, cirrhosis, and hepatocellular carcinoma (HCC), etc. [1]. The causes of CLDs are complex, including hepatic viruses, excessive alcohol consumption, metabolic syndrome, and drug toxicity, which are the major risk factors resulting in chronic liver injury [2]. CLD patients always have persistent inflammation, massive cell death (such as apoptosis and ferroptosis), and abnormal hepatocyte regeneration in the liver, which develop to end-stage liver pathologies, such as cirrhosis and HCC [3]. Due to the increase in hospitalized CLD patients, the economic and social burden has significantly increased, especially in developing countries [4].

Phytochemicals refer to active substances derived from plants, such as polysaccharides, polyphenols, and alkaloids. Many studies have shown that many phytochemicals, such as baicalin and curcumin, have remarkable anti-tumor efficacies with lower side effects compared to other chemotherapeutic drugs [5]. Some phytochemicals have advantageous effects on obesity, cardiovascular diseases, neurological diseases, and cancer by alleviating oxidative stress due to their antioxidative activity [6]. Meanwhile, various natural antioxidants protect against the hepatotoxicity induced by the chemotherapeutic drug cisplatin via antioxidant, anti-inflammatory, and anti-apoptosis activities [7].

The natural sources of polysaccharides are abundant, including plants, fungus, and algae. Polysaccharides have a variety of biological and pharmacological activities, especially in treating diseases, which have been summarized in several recent reviews. For example, polysaccharides have been reported to play protective roles in metabolic syndrome, cardiovascular diseases, and neurodegenerative diseases due to their activities in glucose and lipid metabolism regulation and antioxidant and anti-inflammation activities [8,9,10,11,12]. Other studies revealed algal polysaccharides killing tumor cells via apoptosis while reducing the adverse effect of chemotherapy [13]. Besides, non-starch polysaccharides may improve health by regulating gut microbiota [14]. Moreover, polysaccharides deriving from traditional Chinese medicinal herbs have anti-hypertensive and cardioprotective activities and they also could be used as drug delivery systems to improve therapeutical effects by promoting bioavailability and reducing toxicity [15,16]. Two or three years ago, Yuan et al. summarized the protective effects of polysaccharides in several types of liver injuries [11], and Qu et al. reviewed the signaling pathways by which the plant polysaccharides regulate apoptosis and inflammation [12]. These previous reviews provided insights into the use of polysaccharides in treating liver diseases.

Due to the rapidly increasing number of bioactive phytochemicals, the functions and mechanisms of polysaccharides with hepaprotective effects identified in the latest two years have not been systematically summarized. In this review, we summarize the regulatory functions and mechanisms of various polysaccharides in different liver diseases, including NAFLD, ALD, fibrosis, drug-induced liver injury, and HCC, mainly involving research from the last five years. Moreover, we also summarize the polysaccharides and other types of phytochemicals with activities in regulating ferroptosis, which is the novel mechanism in many types of liver diseases. The progress of studies on polysaccharides and ferroptosis-related phytochemicals will provide novel therapeutic strategies in treating CLDs.

## 2. Polysaccharides in Different Liver Diseases

### 2.1. Nonalcoholic Fatty Liver Disease and Ethanol-induced Liver Disease

Superfluous fatty-acid-induced oxidative stress and inflammation during metabolism are central to the pathogenesis of NAFLD [17]. NAFLD includes simple steatosis and nonalcoholic steatohepatitis (NASH), which is the most common cause of liver dysfunction and is associated with an increased risk of cardiovascular diseases [18,19]. NAFLD is the most universal liver disease in obesity, metabolic syndrome, and diabetes [20]. Generally, without valid treatment, all kinds of chronic hepatitis will finally progress into end-stage liver diseases, such as cirrhosis or HCC [20]. NAFLD is the fastest increasing cause of HCC in many parts of the world, including the USA and parts of Europe [21]. The underlying mechanisms in the development and progression of NAFLD are complex, including insulin resistance, hormones secreted from the adipose tissue, nutrients, and gut microbiota [22].

Alcohol has wide-ranging effects on the gut and liver, resulting in liver inflammation, oxidative damage, fibrosis, and cirrhosis [23]. Alcohol is considered to be a risk factor for liver cirrhosis and has a significant impact on the mortality of liver cirrhosis [24]. The oxidative damage remains a crucial pathology involved in ethanol-induced liver disease (ALD) [25]. Ethanol-induced liver disease is a negative outcome of excessive drinking of ethanol, with increased reactive oxygen species (ROS) during ethanol metabolism in the liver. ROS promote hepatocyte apoptosis by inducing mitochondrial alterations or necrosis by initiating lipid peroxidation on cell membranes [26,27]. A lot of polysaccharides could be used as therapeutics for ameliorating NAFLD or ALD by modulating macronutrient metabolism and reducing cell apoptosis, inflammation, and oxidative stress (Table 1).

Polysaccharides extracted from pomelo fruitlet (YZW-A), *Ophiopogon japonicus* (MDG-1), *Enteromorpha prolifera*, *Schisandra chinensis caulis* (SCP), Chicory (CP), *Ganoderma lucidum* (GLP), *Lycium barbarum* (LBP), *Coriolus versicolor* mycelia (CVMP), *Bletilla striata*, *Cordyceps sinensis* (CSP), mussel polysaccharide α-d-glucan (MP-A), and fucoidan–fucoxanthin mix (FFM) could ameliorate hepatic lipid levels by modulating lipid metabolism [28,29,30,31,32,33,34,35,36,37,38,39,40,41]. Adiponectin reduces hepatic lipid accumulation via AMPK (AMP-activated protein kinase) signaling, which activates lipid oxidation and inhibits fatty acid synthesis [42,43]. YZW-A, MDG-1, CP, MP-A, and CSP could inhibit lipid accumulation in the liver by activating the AMPK pathway [28,35,36,37,40]. CP could significantly reduce hepatic lipid accumulation via increasing the lipid-oxidation-related gene *Pparab*’s expression and reducing lipid-synthesis-related gene (i.e., *Fasn* and *Srebf1*) expression in rats [30,39]. LBP and CVMP could activate the AMPK signaling pathway to reduce steatosis in alcohol-induced fatty liver [33,34].

Many polysaccharides could play hepatoprotective roles in NAFLD via moderating glucose metabolism, such as *Angelica sinensis* polysaccharide (ASP), SCP, and FFM. The PI3K/Akt pathway mediates glucose metabolism to decrease lipid accumulation in the liver [44]. ASP reduced blood glucose levels and ameliorated insulin resistance by activating the PI3K/Akt pathway in high-fat-diet-fed mice [45]. SCP alleviated insulin resistance by regulating the metabolism of ascorbic acid and uronic acid as well as the transformation pathway of pentose and glucuronic acid [29]. FFM has the potential to reduce insulin resistance in patients with NAFLD in a clinical trial [32].

Acidic polysaccharides from carrot (CPS), polysaccharide from the residue of *Panax notoginseng* (PNPS), and modified polysaccharides from *Coprinus comatus* (MPCC) could regulate alcohol metabolism in the liver to reduce hepatic steatosis with the upregulation of hepatic alcohol dehydrogenase and aldehyde dehydrogenase [46,47,48]. *Dendrobium huoshanense* polysaccharide also protected liver function from alcoholic injury via correcting the hepatic methionine disorder [49]. LBP, *Dendrobium officinale* polysaccharide (DOP), *Echinacea purpurea* polysaccharide (EPP), CPS, polysaccharide from *Pleurotus geesteranus* (PFP-1), *Pinus koraiensis* pine nut polysaccharide (PNP80b-2), PNPS, CVMP, MPCC, alkalic-extractable polysaccharides from *Coprinus comatus* (APCC), garlic polysaccharide (GP), *Triticum aestivum* sprout-derived polysaccharide (TASP), and polysaccharide from maca (*Lepidium meyenii*) (MP) were reported to ameliorate alcohol-induced hepatic oxidative stress and inflammatory damage [33,34,46,48,50,51,52,53,54,55,56,57,58,59]. DOP, EPP, PFP-1, PNP80b-2, and TASP could ameliorate alcohol-induced hepatic oxidative stress via promoting the transcription of antioxidant genes mediated by nuclear factor E2-related factor 2 (Nrf2) [50,51,52,54,56]. Polysaccharides from *Pleurotus geesteranus* mycelium, LBP, DOP, and EPP could ameliorate alcohol-induced hepatic inflammatory damage by inhibiting nuclear factor kappa-B (NF-κB) signaling pathways [50,56,60] or by reducing-thioredoxin interacting protein (TXNIP)-induced NLRP3 inflammasome formation [55,56]. Besides, EPP, CPS, PFP-1, MPCC, APCC, *Pleurotus geesteranus mycelium* polysaccharide, GP, TASP, and MP could reverse ethanol-induced lipid disorder, i.e., decreasing serum triglycerides, total cholesterol, and low-density lipoprotein cholesterol (LDL-C) and increasing serum high-density lipoprotein cholesterol (HDL-C) [46,48,50,51,53,54,55,57,58,59].

### 2.2. Hepatic Fibrosis

Hepatic fibrosis is an outcome of wound healing in response to chronic liver injury. Without timely and valid treatment, liver fibrosis might finally develop into end-stage cirrhosis. The mechanisms of liver fibrosis are complex, consisting of inflammation, hepatic stellate cell (HSC) activation, extracellular matrix (ECM) production, and the deposit of collagen in liver [61,62]. Therefore, liver fibrosis can be reversed via ceasing chronic liver damage, blocking inflammation, deactivating HSCs, and degrading ECM [63]. The progression of hepatic fibrosis could be blocked by polysaccharides (Table 1) via these anti-fibrosis pathways.

*O. lanpingensis* polysaccharides (OLP) and *Dictyophora* polysaccharides could significantly decrease the accumulation of ECM and collagen by upregulating MMPs and collagenase expression, which are essential for collagenolysis [64,65]. Quiescent HSCs play important roles in the progression of liver fibrosis because active HSC can transdifferentiate into myofibroblasts, which produce ECM [62]. ASP could alleviate liver fibrosis by activating the IL-22/STAT3 pathway in HSCs to inhibit the HSC–myofibroblast switch [66,67].

In chronic liver damage, the persistent activation of NF-κB signaling and inflammatory cytokines always results in fibrosis [68]. OLP could alleviate liver fibrosis by decreasing inflammatory cytokines and oxidative stress [64], and *Pleurotus citrinipileatus* polysaccharide could inhibit the progression of liver fibrosis via targeting the NF-κB pathway [69].

Intestinal dysbiosis from alcohol or a high-fat diet might result in liver inflammation and fibrosis and eventually develop to liver cirrhosis [70]. Polysaccharides could affect the development of liver fibrosis by improving gut health. LBP, *Miltiorrhiza bunge* polysaccharides, walnut green husk polysaccharides (WGHP), and MDG-1 were reported to alleviate hepatic steatosis via modulating gut microbiota in a high-fat-diet-induced NAFLD model [35,71,72,73]. In a randomized controlled trial, LBP could alleviate the hepatic injury and intestinal dysbiosis in NAFLD patients [74]. WGHP and MDG-1 could moderate the intestinal microecology in mice to reduce liver lipid accumulation [35,72]. Moreover, EPP could attenuate intestinal inflammation and improve barrier integrity to protect against alcohol-induced liver damage [50]. Similarly, DOP also protected against CCl_4_-induced liver fibrosis by improving the intestinal barrier [75].

### 2.3. Hepatocellular Carcinoma (HCC)

Liver cancer is one of the top 10 cancer types, with the mortality of 8.2%, and it ranks fifth in terms of global cases and second in terms of deaths for males. Hepatitis viruses (such as HBV and HCV), alcohol, metabolic syndrome, diabetes, obesity, NAFLD, tobacco, aflatoxins, and other dietary factors have been consistently associated with the effected risk of liver cancer. The prevalence of NAFLD/NASH is increasing and may soon overtake viral factors as the major cause of HCC globally [76]. Several polysaccharides were reported to have therapeutic effects on HCC (Table 1).

Polysaccharides could inhibit the progression of tumors by reducing immunosuppression. The liver has a complex immune microenvironment, and immunosuppressive cells in the tumor tissue can promote HCC tolerance. Tumor-associated macrophages (TAMs), which are one of the key components maintaining the immunosuppressive microenvironment of HCC, can facilitate tumor growth [77]. Therefore, remodeling the microenvironment of tumors could be a therapeutic strategy for anti-tumor immune responses [76]. *Astragalus* polysaccharides (APS) and polysaccharide from *Pleurotus ostreatus* could inhibit HCC growth via immunoregulation with the enhanced secretion of immune-stimulating cytokines (IL-2, TNF-α, IFN-γ, etc.) [78,79]. *Ganoderma lucidum* spore polysaccharide (GLSP) promoted the polarization of primary macrophages into M1 type and cytokine expression (such as TNF-α, IL-1β, IL-6, and TGF-β1) [80].

HCC is highly vascularized. Polysaccharides could inhibit the invasion of HCC cells by reducing tumor angiogenesis. The initiation of angiogenesis is driven by the metabolic demands of tumor cells, such as hypoxia or nutrients. Many factors stimulate this process, including hypoxia-inducible factors (HIFs), mammalian target of rapamycin (mTOR), and PI3K/AKT signaling [81]. Several polysaccharides could block HCC angiogenesis by downregulating hypoxia-inducible factor 1α (HIF-1α) and vascular endothelial growth factors (VEGFs). Moreover, asparagus and dandelion polysaccharides could inhibit MAPK and PI3K signaling pathways to block tumor angiogenesis [82,83,84].

APS, GLSP, fucoidan, *Pleurotus ostreatus* polysaccharide, ginger polysaccharide, *Aconitum coreanum* polysaccharide, pumpkin polysaccharide (PPPF), *Rhizopus Nigrum* polysaccharide, and an acid-soluble polysaccharide from *Grifola frondose* could inhibit hepatocellular carcinoma growth by apoptosis [78,79,80,85,86,87,88,89,90]. The JAK/STAT, PI3K/AKT, and RAS/ERKs pathways are enhanced in many HCC cells, conferring on them resistance to apoptotic stimuli [91]. GLSP triggers HCC cell apoptosis via regulating the PI3K/AKT pathway, with increased Bax/caspases and decreased Bcl-2 [80]. PPPF treatment induces apoptosis in HepG2 cells by increasing the protein tyrosine phosphatase SHP-1 to inhibit JAK2/STAT3 signaling [88].

Many polysaccharides could enhance the effect of chemotherapeutics or simultaneously reduce the negative effects or toxicities of these drugs. Mannan conjugation could enhance the effect of adenovirus-mediated phosphatase and tensin homologue (*PTEN*) gene therapy in a murine HCC model [92]. Polysaccharides from *Lachnum* sp. (LSP) combined with 5-fluorouracil or cyclophosphamide (CTX) and polysaccharides from *Lentinus edodes* combined with oxaliplatin were reported to inhibit the migration and invasion of HCC in a synergistic manner in vitro or in vivo [93,94,95]. Neutral polysaccharide from *Panax notoginseng* combined with CTX and aconitine combined with crude monkshood polysaccharide enhanced the tumor-killing effect by immunoregulation [96,97]. Moreover, nanoparticles made by polysaccharides are also applied in chemotherapeutic drug delivery. ASP, a plant polysaccharide with good biocompatibility, aqueous solubility, and intrinsic liver-targeted capability has been developed into targeted drug delivery nanoparticles for HCC therapy [98,99].

### 2.4. Drug-Induced Liver Injury (DILI)

Due to the first-pass effect of the liver in gastrointestinal nutrition metabolism, the liver is more susceptible to drug toxicity during clinical treatment. The incidence of DILI was estimated to be higher in Asia than that in Western countries [100]. Polysaccharides have significant protective roles in drug-induced liver damage (Table 1). ASP, *Schisandra chinensis acidic* polysaccharide, *Phellinus linteus* mycelia polysaccharide, PNP80b-2, fucoidan, and Seabuckthorn berry polysaccharide could protect against acetaminophen (APAP)-induced acute liver injury and cell death by suppressing oxidative stress [52,101,102,103,104,105]. *Sagittaria sagittifolia* L. polysaccharide and Yulangsan polysaccharide exert protective effects against isoniazid- or rifampicin-induced liver injury via Nrf2 activation and downstream antioxidant gene transcription [106,107]. Meanwhile, the administration of GLP reversed Bacillus Calmette Guérin-induced hepatic injury in vivo via inhibiting nitric oxide production and inflammation [108].

**Table 1:** Polysaccharides in liver diseases.

Polysaccharide	Source	Types of Liver Disease Treated	Cell/Animal Models	Effects and Mechanisms	References
Acidic polysaccharides from carrot (CPS)	Carrot	ALD	Mice	Reducing lipid droplets	[46]
*Aconitum coreanum* polysaccharide	*Aconitum coreanum*	HCC	H22 cells/mice	Inducing apoptosis by suppressing P13K/Akt and activating p38	[86]
alkalic-extractable polysaccharides from *Coprinus comatus* (APCC)	*Coprinus comatus*	ALD	Mice	Inhibiting inflammation and ROS. Improving alcohol metabolism.	[58]
*Angelica sinensis* polysaccharide (ASP)	The dry roots of *Angelica sinensis*	NAFLD	Mice	Inhibiting ROS. Increasing PPARγ and SIRT1-AMPK signaling.	[45]
		Hepatic fibrosis	Mice	Inhibiting inflammation. Decreasing ECM accumulation	[66]
		HCC	Mice	Drug delivery nanoparticles	[98,99]
		DILI	Hepatocytes/rats	Inhibiting ROS and apoptosis	[101]
*Asparagus* polysaccharide	*Asparagus*	HCC	SK-Hep1 and Hep-3B cells	Suppressing MAPK/PI3K and HIF-1α/VEGF signaling pathway	[83,84]
*Astragalus* polysaccharides (APS)	*Astragalus*	HCC	Mice	Inducing apoptosis by increasing Bax and decreasing Bcl-2	[79]
*Bletilla striata* polysaccharide	*Bletilla striata*	NAFLD	Mice	Regulating fatty acids and arachidonic acid metabolism	[41]
Chicory polysaccharide (CP)	Chicory	NAFLD	Zebrafish and rats	Inhibiting ROS and lipogenesis. Promoting lipolysis and AMPK.	[30,37,39]
*Cordyceps sinensis* polysaccharide (CSP)	*Cordyceps* *Sinensis*	NAFLD	Mice	Modulating lipid metabolism and gut microbiota	[28]
*Coriolus versicolor* mycelia polysaccharide (CVMP)	*Coriolus versicolor* mycelia	ALD	Mice	Inhibiting inflammation and ROS. Regulating lipid metabolism	[34]
Crude monkshood polysaccharide	Monkshood	HCC	Hepa1-6 cells/mice	Enhancing the immunocyte to kill the tumor	[97]
Dandelion polysaccharide	Dandelion	HCC	HepG2, Hepa1-6, H22 cells/mice	Suppressing the HIF-1α/VEGF signaling pathway	[82]
*Dendrobium huoshanense* polysaccharide (DHP)	*Dendrobium huoshanense*	ALD	Mice	Correcting the abnormal hepatic methionine metabolism pathway and decreasing the hepatic methylglyoxal level	[49]
*Dendrobium officinale* polysaccharide (DOP)	*Dendrobium officinale*	ALD	L02 cells/rats	Inhibiting TLR4/NF-κB signaling	[56]
		Hepatic fibrosis	Rats	Inhibiting the TLR4-NF-κB pathway	[75]
*Dictyophora* polysaccharides	*Dictyophora*	Hepatic fibrosis	Rats	Decreasing ECM accumulation	[65]
*Echinacea purpurea* polysaccharide (EPP)	*Echinacea purpurea*	ALD	Mice	Activation of the Nrf2/HO-1 pathway	[50]
*Enteromorpha prolifera* polysaccharide	*Enteromorp-ha prolifera*	NAFLD	Rats	Reducing serum lipid levels by increasing H_2_S production	[31]
Fucoidan	Brown algae	HCC	MHCC97H, Hep3B cells/mice	Inducing apoptosis by increasing lncRNA LINC00261 expression	[87]
		DILI	HL7702 cells/mice	Inhibiting ROS by Nrf2 signaling	[105]
Fucoidan–fucoxanthin mix (FFM)	*Sargassum hemiphyllum*	NAFLD	HepaRG cells/mice/patients	Inhibiting inflammation. Modulating the leptin–adiponectin axis	[32]
*Ganoderma lucidum* polysaccharide (GLP)	*Ganoderma lucidum*	NAFLD	HepG2 cells/mice	Modulating bile acid synthesis through the FXR-SHP/FGF pathway	[38]
		DILI	Mice	Inhibiting nitric oxide production and inflammation	[108]
*Ganoderma lucidum* spore polysaccharide (GLSP)	The spores of *Ganoderma lucidum*	HCC	Mice	Promoting the polarization of primary macrophages to the M1 type	[80]
Garlic polysaccharide (GP)	Garlic	ALD	Mice	Regulating gut microbiota	[59]
Ginger polysaccharide	Ginger	HCC	HepG2 cells	Inducing apoptosis	[89]
*Grifola frondose* polysaccharide	*Grifola frondosa*	HCC	H22 and HepG2 cells	Inducing the mitochondrial apoptotic pathway	[90]
*Lycium barbarum* polysaccharide (LBP)	*Lycii Fructus*	NAFLD	Rats/humans	Inhibiting inflammation and regulating host gut microbiota	[71,74]
		ALD	BRL-3A cells/mice	Inhibiting TXNIP and activating AMPK. Inhibiting inflammation, ROS, and apoptosis.	[33,55]
*Miltiorrhiza bunge* polysaccharide	*Salvia miltiorrhiza*	NAFLD	Mice	Modulating gut microbiota and improving insulin resistance	[73]
Modified polysaccharides from *Coprinus comatus* (MPCC)	*Coprinus comatus*	ALD	Mice	Inhibiting inflammation and ROS. Reducing serum lipid levels. Promoting alcohol metabolism.	[48]
Mussel polysaccharide α-D-glucan (MP-A)	*Mytilus coruscus*	NAFLD	Rats	Inhibiting inflammation. Increasing short-chain fatty acids. Inhibiting PPAR signaling.	[36]
Neutral polysaccharide from *Panax notoginseng*	*Panax notoginseng*	HCC	Mice	Enhancing the anti-tumor effect of cyclophosphamide	[96]
*O. lanpingensis* polysaccharides (OLP)	*Ophiocordyceps lanpingensis*	Hepatic fibrosis	Mice	Inhibiting inflammation, ROS, and apoptosis	[64]
*Ophiopogon japonicus* polysaccharide (MDG-1)	*Ophiopogon*	NAFLD	Mice	Inhibiting inflammation. Modulating the gut–liver axis and hepatic lipid metabolism.	[35]
*Phellinus linteus* mycelia polysaccharide (PL-N1)	*Phellinus linteus* mycelia	DILI	Mice	Decreasing cytochrome P450 2E1 expression and hepatic release of cytokines	[103]
*Pinus koraiensis* pine nut polysaccharide (PNP80b)	Pine nut	ALDDILI	Mice	Inhibiting inflammation and ROS by Nrf2 signaling	[52]
*Pleurotus citrinipileatus* polysaccharide	*Pleurotus citrinipileatus*	Hepatic fibrosis	Mice	Reducing the level of cytokine TGF-β1	[69]
Polysaccharide from *Lachnum* sp. (LSP)	*Lachnum* sp.	HCC	HepG2, SMMC7721, H22 and L02 cells/mice	Inducing apoptosis by inhibiting the MEK and PI3K pathways	[94,95]
Polysaccharide from *Lentinus*	*Lentinus edodes*	HCC	HepG2 and H22 cells/mice	Inducing the mitochondrial apoptotic pathway and inhibiting NF-κB, Stat3, and survivin signaling	[93]
Polysaccharide from Maca (MP)	Maca (*Lepidium meyenii*)	ALD	HepG2 cells/ mice	Reducing ROS and serum lipid levels	[57]
Polysaccharide from *Pleurotus geesteranus* mycelium	The mycelium of *Pleurotus geesteranus*	ALD	Mice	Inhibiting inflammation and ROS. Regulating alcohol metabolism. Reducing serum lipid levels.	[53,60]
Polysaccharide from *Pleurotus geesteranus* (PFP-1)	The fruiting body of *Pleurotus geesteranus*	ALD	Mice	Activating Nrf2 signaling and inhibiting the TLR4-mediated NF-κB signal pathways	[54]
Polysaccharide from *Pleurotus ostreatus*	*Pleurotus ostreatus*	HCC	HepG2 and HCCLM3 cells/ mice	Inducing apoptosis. Downregulation of regenerative genes and secretion of immunological factors.	[78]
Polysaccharide from the residue of *Panax notoginseng* (PNPS)	the residue of *Panax notoginseng*	ALD	Mice	Inhibiting inflammation and ROS by Nrf2 signaling. Reducing serum lipid levels.	[47]
Pomelo fruitlet polysaccharide (YZW-A)	Pomelo fruitlet	NAFLD	Mice	Promoting hepatic AMPK and Nrf2 signaling.	[40]
Pumpkin polysaccharide (PPPF)	Pumpkin	HCC	HepG2 cells	Inducing apoptosis by inhibiting the JAK2/STAT3 pathway	[88]
*Rhizopus Nigrum* polysaccharide	*Rhizopus* *Nigrum*	HCC	HepG2 and Huh7 cells/mice	Inducing apoptosis	[85]
*Sagittaria sagittifolia* L. polysaccharide	The root tubers of *S. sagittifolia*	DILI	Mice	Inhibiting ROS by Nrf2	[107]
*Schisandra chinensis caulis* polysaccharide (SCP)	*Schisandra chinensis Caulis*	DILI	Mice	Inhibiting inflammation, ROS, and apoptosis	[102]
		NAFLD	Rats	Inhibiting ROS. Regulating glucose and lipid metabolism.	[29]
Seabuckthorn berry polysaccharide (SP)	The berries of seabuckthorn (*Hippophae rhamnoides* L.)	DILI	Mice	Inhibiting ROS and apoptosis by Nrf2/HO1/SOD signaling	[104]
*Triticum aestivum* sprout-derived polysaccharide (TASP)	*Triticum aestivum*	ALD	Mice	Inhibiting inflammation, ROS, and apoptosis by Nrf2 signaling. Reducing serum lipid levels.	[51]
Walnut green husk polysaccharides (WGHP)	Walnut green husk	NAFLD	Rats	Improving gut microbiota and short-chain fatty acids	[72]
Yulangsan polysaccharide	The root of *Millettia pulchra*	DILI	Mice	Inhibiting ROS	[106]

## 3. Cell Death in Liver Diseases

Cell death is a critical event for liver injury, often persisting over decades. Long-term or massive dysregulated cell death may develop into severe clinical outcomes. For example, massive hepatocellular death always results in liver failure, while hepatocyte immortalization may cause HCC. Different types of cell death (such as apoptosis, necrosis, autophagy, and ferroptosis) trigger specific pathological responses and promote the progression of liver disease through distinct mechanisms [109]. The discovery of novel modes of cell death has greatly improved our understanding of the development of liver disease. 

### 3.1. Polysaccharides Regulating Apoptosis

Apoptosis is classic cell death, and hepatocyte apoptosis is often considered to be the major mechanism of liver injury over decades. At the molecular level, apoptosis is divided into two major branches, the intrinsic and extrinsic pathways. The extrinsic apoptosis of hepatocytes can be initiated by inflammatory cytokines, which then trigger Fas-dependent death-inducing signaling complex and downstream caspase-8/9 activation [110]. The caspase-9-induced pro-death protein BID–BAX axis is the major link between the intrinsic and extrinsic pathways. In the intrinsic apoptotic pathway, the mitochondrial outer membrane permeabilization by BAX and BAK results in the release of mitochondrial pro-death effectors, such as the hemoprotein cytochrome c, which triggers the formation of the apoptosome and caspase-3/7 activation [110,111]. Cytochrome c is normally bound to cardiolipin, and therefore the oxidation of cardiolipin by ROS also triggers cytochrome c release and downstream apoptotic signaling, including caspase activation and death execution [112].

Many polysaccharides have been identified as apoptosis regulators (Table 1). Polysaccharides with activities to suppress apoptosis can protect against NASH, ALD, and APAP-induced acute liver injury. On the other hand, polysaccharides such as apoptosis agonists can inhibit HCC development by promoting tumor cells apoptosis. 

### 3.2. Polysaccharides and Other Phytochemicals Regulating Ferroptosis

**Figure 1:**
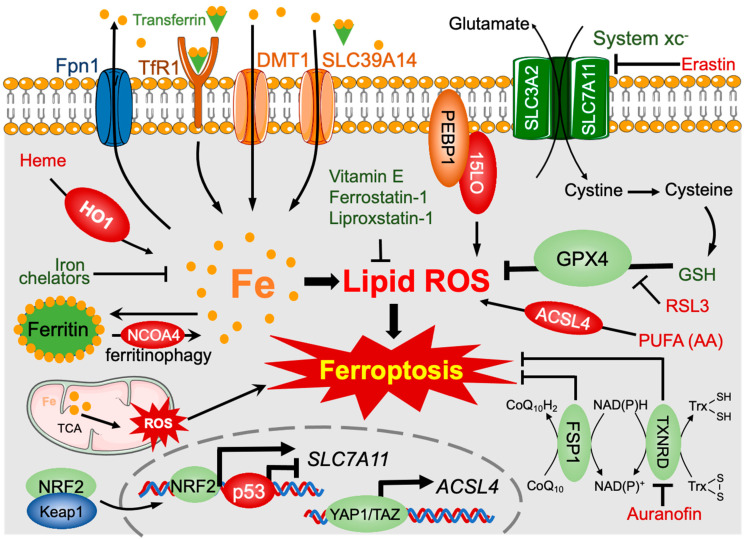
Regulatory pathways of ferroptosis. Iron metabolism is tightly regulated in transport and storage. Cellular iron overload can trigger ferroptosis. Cellular iron uptake is mediated by TfR1, which imports transferrin-binding iron, and by DMT1 and SLC39A14, which import non-transferrin-binding iron. Ferroportin1 (Fpn1) is the only known iron exporter to date. Heme can be degraded by HO-1 to release free iron. Cellular excess iron is stored in ferritin, while ferritin can be degraded by NCOA4-mediated ferritinophagy in an iron-deficiency condition. System xc^−^, a heterodimer composed of SLC7A11 and SLC3A2, is a cystine/glutamate antiporter that mediates the efflux of glutamate and the influx of cystine at a 1:1 ratio. After entering the cell, cystine is reduced to cysteine and then synthesized into GSH. GPX4 scavenges lipid ROS via GSH. Lipid ROS derives from PUFAs-PE oxidation by lipoxygenases. The scaffolding protein PEBP1 can bind PE on the cell membrane and then recruit the lipoxygenase 15LO to generate lipid ROS. ACSL4 can increase lipid ROS by producing PUFAs-PE. Moreover, TCA cycle disorder or iron overload in mitochondria can also increase ROS, which results in ferroptosis. The CoQ/FSP1 and Trx/TXNRD axes inhibit ferroptosis in a GSH-independent manner. The Keap1/NRF2, p53, and YAP/TAZ signaling are necessary for the transcription of ferroptosis regulators, such as *SLC7A11* and *ACSL4*. Erastin, RSL3, and auranofin are ferroptosis agonists by targeting SLC7A11, GPX4, and TXNRD, respectively. Ferroptosis inhibitors include iron chelators and lipid ROS scavengers (ferrostatin-1, liproxstatin-1, vitamin E, etc.).

Ferroptosis is a new type of cell death that was identified in 2012 [113]. Ferroptosis was observed in RAS-mutated tumor cells treated with the lethal compound erastin or RSL3. RAS mutations always result in apoptosis resistance, indicating ferroptosis is morphologically, biochemically, and genetically distinct from other forms of cell death. In the discovery of ferroptosis, either lipid peroxidation scavengers (i.e., ferrostatin-1) or iron chelators (i.e., deferoxamine) could specifically inhibit ferroptosis agonist (erastin or RSL3)-induced cell death, and therefore ferroptosis was characterized as a lipid-peroxidation-induced and iron-dependent cell death [113,114,115]. Ferroptosis serves as a major pathological mechanism in a wide range of organs, including the liver, heart, brain, and kidney [115,116,117]. In the past decade, the regulatory mechanisms of ferroptosis have been revealed (Figure 1) but not fully elucidated. Iron homeostasis is tightly maintained in the body, including iron absorption, storage, and utilization. Dysregulated iron metabolism is the key trigger of ferroptosis. In previous studies, an iron overload resulting from a high-iron diet or hereditary hemochromatosis was shown to cause hepatic ferroptosis, and an iron-deficient diet challenge or ferrostatin-1 treatment could rescue iron-overload-induced ferroptosis and liver damage [118,119]. Moreover, in normal cells, excessive iron is stored in ferritin, and the deletion of ferritin H in cardiomyocytes could increase the liable iron pool and result in ferroptotic heart injury [120]. Lipid peroxidation, the oxygenation of polyunsaturated phosphatidylethanolamines (PEs) in the cytoplasm membrane or mitochondrial membrane, is considered to be the executor of ferroptosis by decreasing the membrane integrity [117,121]. Lipid peroxidation is mediated by PE-binding protein 1 (PEBP1), a scaffolding protein that binds with both PEs and lipoxygenases and allows them to generate lipid peroxides [115]. The antioxidant glutathione (GSH)–glutathione peroxidases (GPXs) axis is a major mechanism for cleaning lipid peroxidation. The cystine/glutamate antiporter xc^−^ is essential for cellular GSH, and its subunit SLC7A11 mediates cystine uptake, which is then reduced into cysteine for GSH synthesis [115]. The genetic deletion or mutation of SLC7A11 inhibited GSH synthesis and resulted in increased tissue lipid peroxidation and ferroptotic injury [118], while overexpressing SLC7A11 could increase the GSH content and rescue ferritin H knockout-induced ferroptotic heart damage [120]. GPX4, an enzyme that catalyzes GSH reacting with lipid peroxidation, plays critical roles in blocking ferroptosis. Therefore, inducing ferroptosis by the pharmacological inhibition of SLC7A11 and GPX4 provides novel strategies for tumor chemotherapy [113,122,123,124]. Nrf2 is the key transcription factor of many antioxidant genes involved in ferroptosis, including *SLC7A11* and *GPXs*. Besides GSH, other antioxidants, including NADPH and reduced thioredoxin (Trx), can also inhibit ferroptosis by reducing lipid peroxidation [125,126,127]. Recently, the FDA-approved anti-rheumatoid arthritis drug auranofin was identified as a a novel ferroptosis agonist by pan-inhibiting thioredoxin reductases (TXNRDs), which could refresh reduced Trx after reacting with lipid peroxidation. Therefore, auranofin and ferroptosis inhibitor (i.e., ferrostatin-1) combined treatment was suggested to be a safer strategy in the clinic to avoid ferroptotic toxicity from high-dose auranofin [127]. Moreover, polyunsaturated fatty acids (PUFAs) are essential for ferroptosis due to their sensitivity to lipid peroxidation [128,129]. ACSL4, an enzyme that catalyzes arachidonic acids synthesizing into PUFAs, could drive ferroptosis via oxidized phospholipids accumulating in the cell membrane [130,131,132,133]. 

Several polysaccharides have been identified as ferroptosis regulators to date, consisting of ferroptosis agonists and inhibitors (Table 2). Red ginseng polysaccharide and LBP exhibited anti-tumor efficacy by triggering ferroptosis [134,135,136]. Fucoidans, APS, and polysaccharide of *atractylodes macrocephala Koidz* could alleviate tissue injuries by inhibiting ferroptosis [137,138,139].

The liver is one of the most important organs for iron storage. The hepatic iron and ROS burden are greater in the diseased liver than in the normal liver, suggesting that ferroptosis may be associated with chronic liver diseases [116]. Currently, ferroptosis has been identified as the key mechanism in NASH, ALD, ischemia/reperfusion, and iron overload (hemochromatosis)-related liver injury [118,140,141,142,143]. However, polysaccharides have not been reported to regulate ferroptosis in liver diseases, while many phytochemicals of other types, such as terpene, alkaloid, and flavonoid, can alleviate the pathogenesis of liver diseases via regulating ferroptosis (Table 2). For one thing, phytochemicals could induce ferroptosis to suppress the progression of liver fibrosis and HCC. For instance, magnesium isoglycyrrhizinate, derivatives of artemisinin (such as artemether, artesunate, and dihydro-artemisinin (DHA)), wild bitter melon extracts, chrysophanol, and zalkaloid berberine could block the development of liver fibrosis by triggering HSC ferroptosis [144,145,146,147,148,149,150,151,152]. Besides, several studies revealed that DHA could trigger ferroptosis to block HCC growth by promoting PEBP1/15LO formation or an unfolded protein response [153,154]. Moreover, DHA and artesunate could enhance the anti-tumor efficacy of sorafenib on HCC by inducing ferroptosis [155,156]. In addition, alkaloid solasonine promotes the ferroptosis of HCC cells via inhibiting GPX4 and GSH synthetase [157]. Meanwhile, heteronemin, a natural marine product isolated from *Hippospongia sp.*, was reported to trigger HCC cell ferroptosis and apoptosis by increasing intracellular ROS and inhibiting MAPK signaling [158].

For another thing, some phytochemicals also inhibit hepatic ferroptosis to protect against NASH, drug-induced liver injury, and acute liver failure (Table 2). For example, some investigations discovered that some natural products, such as ginkgolide B and dehydroabietic acid, could alleviate NASH pathology by activating Nrf2 signaling to inhibit ferroptosis [159,160]. Clausenamide could prevent drug-induced hepatocyte ferroptosis via the activation of the Keap1-Nrf2 pathway [161]. Glycyrrhizin significantly reduced the degree of ferroptosis in acute liver failure by enhancing glutathione synthesis [162]. Holly (*Ilex latifolia* Thunb.) polyphenol extracts are able to relieve hepatic ferroptosis by inhibiting iron transport and enhancing GPX4 expression [163]. Moreover, baicalein supplementation ameliorates CCl_4_-induced acute liver injury in mice by inhibiting ferroptosis and inflammation, which involves the activation of Nrf2 and the inhibition of lipoxygenases and the NF-kB pathway [164].

**Table 2:** Phytochemicals in ferroptotic diseases.

Agonist/Inhibitor	Phytochemicals	Types of Phytochemicals	Types of Diseases Treated	Cell/Animal Models	Mechanisms	References
Agonist	Artemether	Terpene	Liver fibrosis	LX2 cells/mice	Activiting p53 signaling. Accumulating IRP2	[148,151]
Agonist	Artesunate	Terpene	Liver fibrosis	Mice	Promoting ferritinophagy	[145]
			HCC	Huh7, SNU-449, SNU-182 HCC cells	Promoting ferritin degradation and decreasing GSH	[156]
Agonist	Chrysophanol	Quinone	Liver fibrosis	Mice	Promoting ER stress	[146]
Agonist	Dihydroartemisinin (DHA)	Terpene	Liver fibrosis	Rats, mice	Promoting ferritinophagy	[150,152]
			HCC	Hep3B, HepG2, and Huh7 cells/mice	Promoting ER stress and PEBP1/15-LO formation	[153,154,155]
Agonist	Heteronemin	Terpene	HCC	HA22T, HA59T cells	Increasing ROS	[158]
Agonist	*Lycium barbarum* polysaccharide (LBP)	Polysaccharide	Breast cancer	MCF-7 and MDA-MB-231 cells	Triggering ferroptosis by downregulating SLC7A11 and GPX4	[134]
Agonist	Magnesium isoglycyrrhizinate	Terpene	Liver fibrosis	Rats	Increasing HO-1 expression	[147]
Agonist	*Red ginseng* polysaccharide	Polysaccharide	Lung and breast cancer	A549 and MDA-MB-231 cells	Triggering ferroptosis by inhibiting GPX4	[136]
Agonist	Solasonine	Alkaloid	HCC	HepG2, HepRG cells	Inhibiting GPX4 and GSH synthetase	[157]
Agonist	Wild bitter melon extract		Liver fibrosis	Mice	Inhibiting GPX4 and SLC7A11	[144]
Agonist	Alkaloid berberine	Alkaloid	Liver fibrosis	Mice	Blocking the autophagy–lysosome pathway and increasing ROS	[149]
Inhibitor	*Astragalus* polysaccharide (APS)	Polysaccharide	Colitis	Caco-2 cells/DSS-challenged mice	Decreasing lipid ROS	[137]
Inhibitor	Baicalein	Flavonoid	Acute liver injury	HepG2 cells/mice	Inhibiting the NF-κB pathway and ALOX12	[164]
Inhibitor	Clausenamide	Pyrrolidone	DILI	Hepa RG and HepG2 cells/mice	Activating the Keap1-Nrf2 pathway	[161]
Inhibitor	Dehydroabietic acid	Terpene	NAFLD	HEK293T and HL7702 cells/mice	Activating the Nrf2-ARE pathway	[159]
Inhibitor	Fucoidans	Polysaccharide	Retinal disease	ARPE-19 and OMM-1 cells	Inhibiting ferroptosis by increasing GPX4	[138]
Inhibitor	Ginkgolide B	Terpene	NAFLD	HepG2 cells/ mice	Activating Nrf2 signaling	[160]
Inhibitor	Glycyrrhizin	Terpene	Acute liver injury	L02 cells/mice	Promoting the Nrf2/HO-1/HMGB1 pathway	[162]
Inhibitor	Holly (*Ilex latifolia* Thunb.) polyphenols	Polyphenol	Acute liver injury	Piglet	Decreasing lipid ROS	[163]
Inhibitor	Polysaccharide of *atractylodes macrocephala Koidz*	Polysaccharide	Spleen injury in infections	Goslings	Inhibiting ferroptosis by restoring the expression and distribution of GPX4	[139]

## 4. Conclusions and Future Directions

Liver disease is a global health burden that has complex mechanisms and needs effective therapeutics in the early stage of liver injury. Given a growing number of polysaccharides with bioactivities (such as antioxidant, immunoregulation, and tumor killing activities) have been identified, the polysaccharide may provide a promising therapeutic strategy for liver diseases. Recently, various natural polysaccharides have been reported to possess protective roles in several liver diseases resulting from fatty liver, alcohol, drug toxicity, or HCC. Moreover, angelica polysaccharides can be developed into a hypoxia-responsive nano-drug delivery system that facilitated HCC chemotherapy. However, studies about polysaccharides on virus hepatitis have been reported less than other liver diseases, suggesting polysaccharides with anti-virus bioactivity need to be identified. Currently, the majority of data are collected from in vitro and animal experiments. Therefore, further studies in humans are needed in order to evaluate the efficacy of these polysaccharides in the clinic.

Cell death, including apoptosis or ferroptosis, is a double-edged sword for health. Therefore, phytochemicals, such as cell death agonists or inhibitors, may play different roles in treating liver diseases. For example, some phytochemicals that inhibit cell death could alleviate ALD, DILI, or chemotherapeutic toxicity in the liver, while lethal phytochemicals may serve as chemotherapeutics in HCC. Ferroptosis is a new type of cell death with features of iron and lipid ROS accumulation that is different from other types of cell death. The discovery of ferroptosis has greatly improved the understanding and therapeutic strategies of liver disease. Several compounds and phytochemicals could alleviate liver injury by targeting ferroptosis, while inhibitors of other cell death (such as apoptosis) could not. Moreover, phytochemicals with ferroptosis-inducing activities might be effective and promising drugs for HCC because ferroptosis agonists can evade the drug resistance of classic chemotherapeutics (e.g., cisplatin, which kills tumors by apoptosis). Therefore, elucidating the mechanisms of ferroptosis and identifying more ferroptosis-regulatory phytochemicals may provide novel therapeutic strategies for liver diseases in the future.

## Data Availability

Not applicable.

## Abbreviations

ALD	alcoholic liver disease
AMPK	AMP-activated protein kinase
APAP	acetaminophen
APCC	alkalic-extractable polysaccharides from *Coprinus comatus*
APS	*Astragalus* polysaccharides
ASP	*Angelica sinensis* polysaccharide
CLD	chronic liver disease
CP	chicory polysaccharide
CPS	carrot polysaccharide
CSP	*Cordyceps sinensis* polysaccharide
CTX	cyclophosphamide
CVMP	polysaccharide from *Coriolus versicolor* mycelia
DHA	dihydroartemisinin
DILI	drug-induced liver injury
DOP	*Dendrobium officinale* polysaccharide
ECM	extracellular matrix
EPP	*Echinacea purpurea* polysaccharide
FFM	fucoidan and fucoxanthin mix
GLP	*Ganoderma lucidum* polysaccharide
GLSP	*Ganoderma lucidum* spore polysaccharide
GP	garlic polysaccharide
GPX	glutathione-glutathione peroxidases
GSH	glutathione
HCC	hepatocellular carcinoma
HDL-C	high-density lipoprotein cholesterol
HIF	hypoxia-inducible factor
HIF-1a	hypoxia-inducible factor 1a
HSC	hepatic stellate cells
LBP	*Lycium barbarum* polysaccharide
LDL-C	low-density lipoprotein cholesterol
LSP	polysaccharide from *Lachnum* sp.
MDG-1	*Ophiopogon japonicus* polysaccharide
MP	maca (*Lepidium meyenii*) polysaccharide
MP-A	mussel polysaccharide α-D-glucan
MPCC	modified polysaccharides from *Coprinus comatus*
mTOR	mammalian target of rapamycin
NAFLD	nonalcoholic fatty liver disease
NASH	nonalcoholic steatohepatitis
NF-κB	nuclear factor kappa-B
Nrf2	nuclear factor E2-related factor 2
OLP	*O. lanpingensis* polysaccharides
PE	phosphatidylethanolamine
PEBP1	PE-binding protein 1
PFP-1	*Pleurotus geesteranus* polysaccharide
PNP80b-2	*Pinus koraiensis* pine nut polysaccharide
PNPS	polysaccharide from the residue of *Panax notoginseng*
PPPF	polysaccharide from pumpkin fruit
PUFAs	polyunsaturated fatty acids
ROS	reactive oxygen species
SCP	*Schisandra chinensis* caulis polysaccharide
TAMs	tumor-associated macrophages
TASP	*Triticum aestivum* sprout-derived polysaccharide
Trx	thioredoxin
TXNIP	thioredoxin-interacting protein
TXNRD	thioredoxin reductase
VEGFs	vascular endothelial growth factors
WGHP	walnut green husk polysaccharides
YZW-A	polysaccharide extract from pomelo fruitlet
